# The biofunction of *Akkermansia muciniphila* in intestinal-related diseases

**DOI:** 10.20517/mrr.2024.12

**Published:** 2024-09-05

**Authors:** Ping Jiang, Siqi Ji, Dan Su, Yu Zhao, Viriania Berta Esperanca Goncalves, Guifang Xu, Mingming Zhang

**Affiliations:** ^1^Department of Gastroenterology, Nanjing Drum Tower Hospital, Affiliated Hospital of Medical School, Nanjing University, Nanjing 210008, Jiangsu, China.; ^2^Division of Gastroenterology and Hepatology, Shanghai Institute of Digestive Disease, NHC Key Laboratory of Digestive Diseases, State Key Laboratory for Oncogenes and Related Genes, Renji Hospital, School of Medicine, Shanghai Jiao Tong University, Shanghai 200001, China.; ^3^FUJIFILM Diosynth Biotechnologies, Watertown, MA 02472, USA.; ^4^University of Chicago, Pritzker School of Molecular Engineering, Chicago, IL 60637, USA.; ^5^Department of Gastroenterology, Nanjing Drum Tower Hospital Clinical College of Nanjing Medical University, Nanjing 211166, Jiangsu, China.; ^#^Authors contributed equally.

**Keywords:** *Akkermansia muciniphila*, intestinal inflammation, colorectal cancer, metabolic disease, probiotic enzyme

## Abstract

Intestinal homeostasis is essential for maintaining human health, and its dysfunction is related to the onset and progression of various diseases, including immune and metabolic disorders, and even tumorigenesis. Intestinal microbiota plays a critical role in intestinal homeostasis, with *Akkermansia muciniphila* (*A. muciniphila*) emerging as a key commensal bacterium utilizing mucin as its sole carbon and nitrogen source. *A. muciniphila* has been recognized in both experimental and clinical studies for its beneficial role in managing intestinal inflammation, tumors, functional gastrointestinal disorders, and secondary conditions such as liver and metabolic diseases. This review provides a comprehensive overview of the research history and current understanding of *A. muciniphila*, its association with various intestinal-related diseases, and the potential mechanisms behind its effects. This paper also explores the possibilities of leveraging the probiotic enzyme such as the active ingredients of *A. muciniphila* for the innovative clinical treatment of intestinal-related diseases.

## INTRODUCTION

The intestinal microbiota is important in maintaining many significant physiological functions of the body, e.g., assisting the host in digesting food and providing nutrients. The occurrence of a variety of diseases is accompanied by the destruction of intestinal microbiota. In recent years, many intestinal diseases have been found to be inextricably linked to intestinal microbiota disorders such as intestinal inflammation, functional gastrointestinal diseases, and intestinal tumors. The development of liver diseases and metabolic syndromes such as diabetes is also accompanied by ecological dysbiosis of the intestinal microbiota associated with the development of intestinal diseases. In light of these factors, probiotics and their derivatives show promise in addressing gut microbiota imbalances and potentially treating a wide array of diseases. Among the identified next-generation beneficial bacteria^[1]^, *Akkermansia muciniphila* (*A. muciniphila*, *Akk*) has been recognized as a particularly promising candidate^[2]^. *Akk* is an anaerobic intestinal bacterium belonging to the *Verrucomicrobiota*, one of the commensal bacteria in the normal intestine. *Akk* was abundant in the mucosal layer of the host intestine, especially in the cecum. It is prevalent in the gut of healthy adults and infants, accounting for 1%-4% of the total intestinal microbiota colonized from early life^[3]^. *Akk* is closely related to the immune response, energy metabolism, microbial composition and intestinal mucin secretion. It may also play an important role in most intestinal diseases such as intestinal inflammation, intestinal tumors, functional gastrointestinal disorders, and other intestinal-related diseases such as diabetes, obesity and liver disease^[4-6]^. This article reviews the research history of *Akk*, its relationship with intestinal-related diseases, and the possible mechanisms, with the aim to help lay the theoretical foundation for further research on its specific treatment mechanisms and provide inspiration for the treatment and prevention of related diseases.

## THE RESEARCH HISTORY OF *A. MUCINIPHILA*

**Figure 1:**
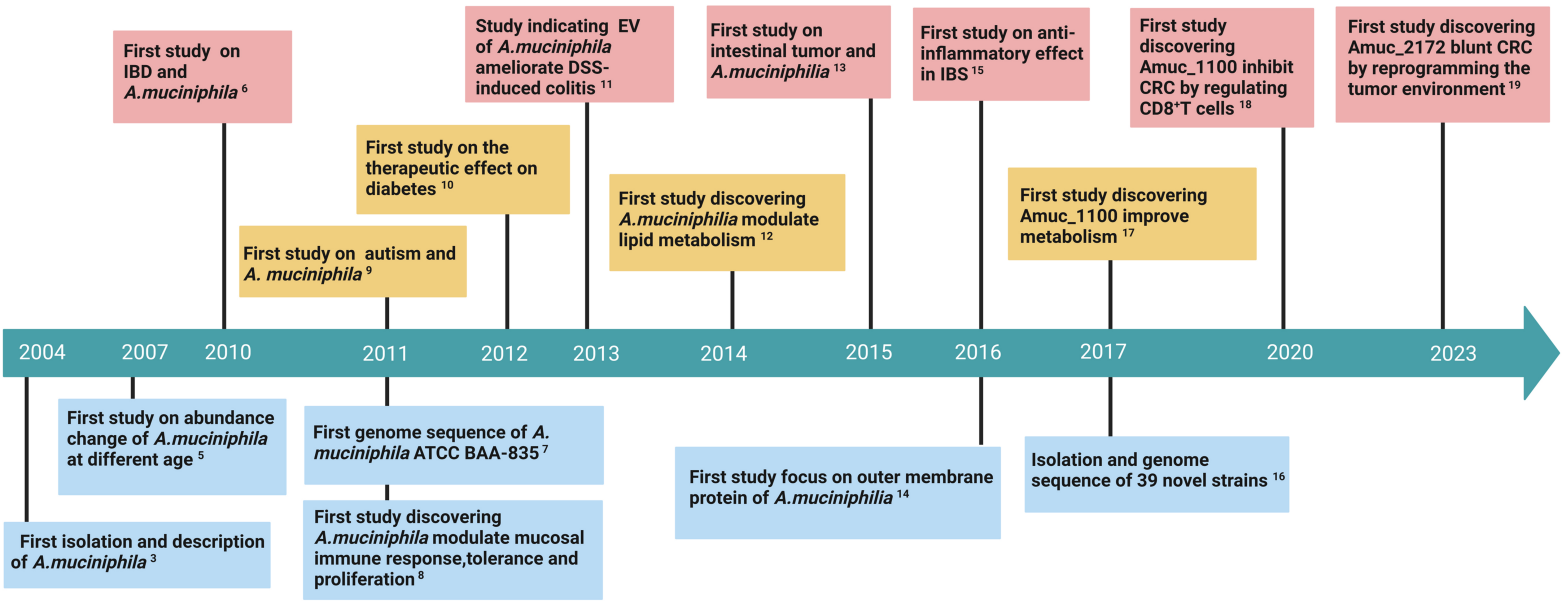
Timeline of significant research milestones in *Akk*. *Akk* was first isolated, identified, and named in 2004. In the first five years, there were few reports on it. After 2010, it gradually received more attention, and research on the relationship between *Akk* and human health and diseases increased significantly. Since 2017, more and more research has focused on the mechanism of interaction between *Akk* and the host to enhance disease management, with particular attention to the proteins and extracellular vesicles of *Akk*. Created with BioRender.com. *Akk*: *Akkermansia muciniphila*.

*Akk* is an anaerobic Gram-negative bacterium that is oval in shape, immobile and does not form endospores. *Akk* belongs to the *Verrucomicrobiota*, *Akkermansiaceae*, and *Akkermansia*^[7]^, and is the first member and the only representative of *Verrucomicrobiota* in the human gut^[8]^. The history of research on *Akk* is not long [Figure 1], with its first isolation and identification in 2004 from human feces via anaerobic culture by researchers from the Microbiology Laboratory of Wageningen University in the Netherlands^[7]^. In the first 5 years after its identification, there was only 1 report on *Akk* each year. Since 2010, *Akk* has been gaining attention with significantly increasing studies on its genomic data and relationship with human health and diseases. In 2007, a study using fluorescence in situ hybridization and real-time PCR analysis for the 16S rRNA gene of *Akk* showed that it can be colonized in the intestine of infants, reach a level close to the abundance in the intestine as healthy adults within 1 year, and gradually decrease in the elderly^[9]^. In 2010, Png *et al.* found that *Akk* abundance was substantially reduced in the intestinal epithelium of Crohn’s disease (CD) and ulcerative colitis (UC) patients^[10]^. Furthermore, researchers sequenced the genome of *Akk* ATCC BAA-835 in 2011 and found that its complete genome was composed of a 2,664,102 bp circular chromosome with an average GC content of 55.8%^[11]^. In the same year, studies found that *Akk* colonization altered mucosal gene expression in germ-free mice, increased the expression of genes involved in immune responses^[12]^, and that the relative abundance of *Akk* was lower in children with autism^[13]^. In 2012, Hansen *et al.* found for the first time that the mucolytic bacterium *Akk* plays a protective role in the development of autoimmune diabetes, especially in the infancy of mice^[5]^. In 2013, *Akk*-derived extracellular vesicles were found to be protective in the development of dextran sulfate sodium salt (DSS)-induced colitis^[14]^. In 2014, studies observed that *Akk* affects the expression of genes involved in host lipid metabolism and epigenetic activation, reduces the risk of gastrointestinal diseases^[15]^, and that the application of *Akk* alters the metabolic characteristics of nonalcoholic fatty liver disease (NAFLD) and obesity, thereby alleviating the disease. In 2015, a study using a colorectal cancer (CRC) mouse model revealed that *Akk* colonization significantly reduced the number of intestinal tumors, goblet cell density, and mucus layer thickness compared to the control group, suggesting that *Akk* treatment plays a beneficial role in suppressing intestinal tumors^[16]^. A 2016 study^[17]^ first identified the outer membrane proteins of *Akk*, and another study found that *Akk* is involved in the anti-inflammatory effects of the intestinal microbiota in constipated irritable bowel syndrome (IBS)^[18]^. In 2017, Guo *et al.* used whole-genome shotgun sequencing to isolate 39 new *Akk* strains from human and mouse fecal samples, followed by sequencing of the total DNA of all microbes, assembly and annotation to identify 5,644 unique proteins^[19]^. Another study found that purified membrane protein Amuc_1100 isolated from *Akk* improved metabolism in diabetic and obese mice^[20]^. In the following five years, an increasing number of studies began to focus on the mechanisms by which *Akk* interacted with the host to ameliorate disease, with particular attention to intestinal-related diseases, as well as proteins and extracellular vesicles of *Akk*. In 2020, Wang *et al.* demonstrated that *Akk* or its purified membrane protein Amuc_1100 could inhibit the occurrence of CRC associated with colitis by regulating CD8^+^ T cells^[21]^. Furthermore, the researchers recently found a new protein of *Akk* named Amuc_2172, which was derived from extracellular vesicles of *Akk*, can serve as acetyltransferase to inhibit the development of CRC by reprogramming the tumor microenvironment, and was also effective in other tumor models, such as melanoma^[4]^. As Amuc_2172 is an enzyme secreted by bacteria that can improve the function of host cells and alleviate the disease process of the host, we name it a probiotic enzyme.

## THE THERAPEUTIC POTENTIAL OF *AKK* IN INTESTINAL-RELATED DISEASES

### *Akk* and intestinal inflammation

**Figure 2:**
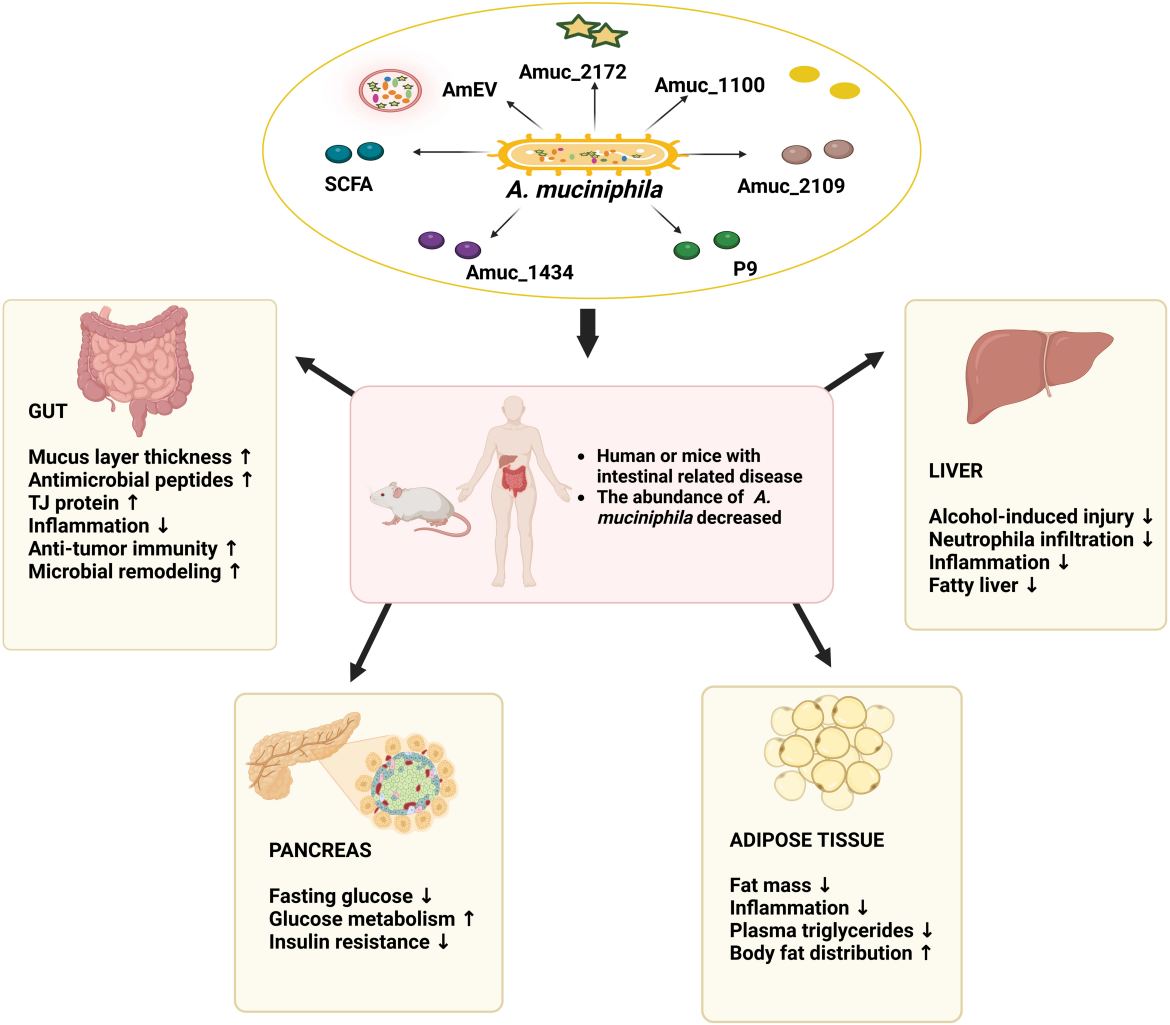
The effect of *Akk* on intestinal-related diseases. Administration of *Akk* or its components to humans or mice can alleviate intestinal inflammation, tumors, and functional gastrointestinal diseases, as well as metabolic regulation such as alleviation of obesity, insulin resistance, and liver disease. Administering *Akk* or its components to humans or mice has been shown to reduce intestinal inflammation, inhibit tumor growth, and alleviate functional gastrointestinal disorders. Additionally, it contributes to metabolic regulation, including the reduction of obesity, improvement of insulin sensitivity, and mitigation of liver disease. Created with BioRender.com. *Akk*: *Akkermansia muciniphila*.

Inflammatory bowel disease (IBD) is influenced by a complex interaction between immune, genetic, environmental factors, and the intestinal microbiota. Multiple studies have shown that intestinal inflammation is associated with changes in the abundance of *Akk*, most of which reveal that *Akk* may play a beneficial role in controlling intestinal inflammation [Figure 2], as the occurrence of intestinal inflammation is often accompanied by the reduction of *Akk*, but some findings are not completely consistent with this. Studies on IBD patients showed that compared with healthy people, the relative abundance of *Akk* in the colon was significantly reduced in UC patients, as well as in CD patients^[10,22-24]^. Several experiments on mouse models of colitis also support a beneficial role for *Akk* in controlling intestinal inflammation. For example, a study in 2020 showed that the abundance of *Akk* was significantly reduced in DSS-induced colitis mice^[21]^. After treatment of DSS-induced colitis mice with secondary bile acid, the abundance of *Akk* in the feces was significantly increased^[25]^. Similar results were obtained with hyaluronic acid-bilirubin nanomedicine (HABN) treatment^[26]^. It was also found in an *Escherichia coli* infectious ileitis model that mice overexpressing tryptophan metabolizing enzyme indoleamine 2,3-dioxygenase 1 (IDO1) showed remission of inflammation, thickened intestinal mucus layer, and increased proportion of *Akk* compared to control mice^[27]^. Furthermore, in an intestinal epithelium-specific autophagy-related protein 5 (ATG5) knockout mouse model, ATG5 deficiency was found to result in a significant reduction in *Akk*^[28]^. Furthermore, extracellular vesicles of *Akk* (AmEV) were significantly reduced in the intestine of mice with DSS-induced colitis^[14]^.

In addition to these correlative studies, researchers have also attempted interventional studies with *Akk* or its components. The application of *Akk* reduced the histopathological score of colitis mice by increasing the thickness of the colonic mucus layer, which proved that *Akk* played a positive role in improving intestinal barrier function and reducing intestinal inflammation^[29]^. Furthermore, a study in 2020 showed that Amuc_1100, the purified membrane proteins of *Akk*, attenuated DSS-induced colitis by reducing colon-infiltrating macrophages and cytotoxic T lymphocytes (CTLs)^[21]^. In addition, Bian *et al.* in 2019 demonstrated that the application of *Akk* improved intestinal inflammation in DSS-induced mice. It reduced the levels of inflammatory cytokines such as TNF-α, IL-6, IL-1α, MIP-1A, IL-12A, and G-CSF, while altering the intestinal microbiota of mice^[30]^. In addition, *in vitro* treatment of colonic epithelial cells with AmEVs reduced the production of IL-6, and oral administration of AmEV to mice alleviated DSS-induced colitis phenotypes such as inflammatory cell infiltration, weight loss, and shortened colonic length^[14]^. Studies exploring the anti-inflammatory properties of two *Akk* strains against DSS-induced chronic colitis in mice also obtained similar results^[29]^.

### *Akk* and intestinal tumors

Many studies have shown that the risk of developing CRC is two to four times higher in patients with IBD than in normal subjects^[31]^. The IBD-CRC model can destabilize the diversity of the intestinal microbiota^[32]^. According to a recent study, intestinal microbiota may be a driver of CRC development. The study proposes a “driver-passenger” model in which the original gut bacteria act as drivers to attack the gut, causing DNA damage and thus inducing CRC. Tumorigenesis causes changes in intestinal microbiota, which creates favorable conditions for the propagation of “passenger” bacteria^[29]^. In this context, the application of probiotics may be a potential strategy to combat intestinal tumors [Figure 2]. In 2013, Grivennikov *et al.* suggested that supplementation with *Akk* could prevent intestinal cancer by attenuating DNA damage and inhibiting abnormal proliferation of tumor cells^[33]^. Another study found that vitamin D supplementation inhibited the development and progression of CRC and increased the integrity of the colonic barrier and the abundance of *Akk*^[34]^, which suggested that *Akk* may be a potential probiotic for the treatment of CRC.

Further studies revealed that *Akk* abundance was significantly reduced in the feces of patients and mice with CRC^[35]^. In addition, *Akk* was found to actively promote the response to chemotherapeutic agents and immune checkpoint inhibitors in tumors^[36]^. In 2017, researchers conducted a trial of 249 patients treated with immune checkpoint inhibitor programmed cell death protein 1 (PD-1) antibodies^[37]^, 69 of whom were also treated with broad-spectrum antibiotics. It was observed that in the group of patients treated with antibiotics, there was a higher probability of tumor recurrence and a shorter survival time. It was found that the presence of *Akk* was associated with more effective immunotherapy in patients not treated with antibiotics. To verify this correlation, the researchers transplanted feces from patients with good immunotherapeutic effects into germ-free mice and found that the immune effects were higher than those of non-transplanted mice. In mice given *Akk* by gavage, IL-12 levels increased and promoted the recruitment of T lymphocytes to mouse tumors, which in turn restored the efficacy of the anti-PD-1 antibody, and the tumors almost completely disappeared^[37]^. This study demonstrates for the first time the role of *Akk* in the tumor immune response. In addition, *Akk* was significantly increased in individuals treated with FOLFOX (oxaliplatin + fluorouracil + calcium folinate), the first-line chemotherapy regimen for colon cancer treatment in clinical practice, and *Akk* was positively correlated with treatment efficacy^[38]^. In addition, it was found that *Akk* can inhibit tryptophan metabolism by inhibiting the AhR/β-catenin signaling pathway, so as to inhibit the progress of CRC^[39]^.

In recent years, the role of the components of *Akk* in intestinal tumors has attracted considerable attention. In an *in vitro* cellular assay in 2020, the *Akk* aspartate protease Amuc_1434 was demonstrated to inhibit the viability of human CRC LS174T cells^[40]^. Furthermore, oral administration of pasteurized *Akk* or its purified membrane protein Amuc_1100 increased the number of CTL in the colon and mesenteric lymph nodes (MLN) and inhibited azoxymethane (AOM)/DSS-induced colitis and CRC in mice^[21]^. Recently, extracellular vesicles from *Akk* were shown to inhibit the development of CRC associated with colitis. The researchers identified a new protein from AmEV, named Amuc_2172, that promotes the activity of CTL to inhibit CRC^[4]^.

**Figure 3:**
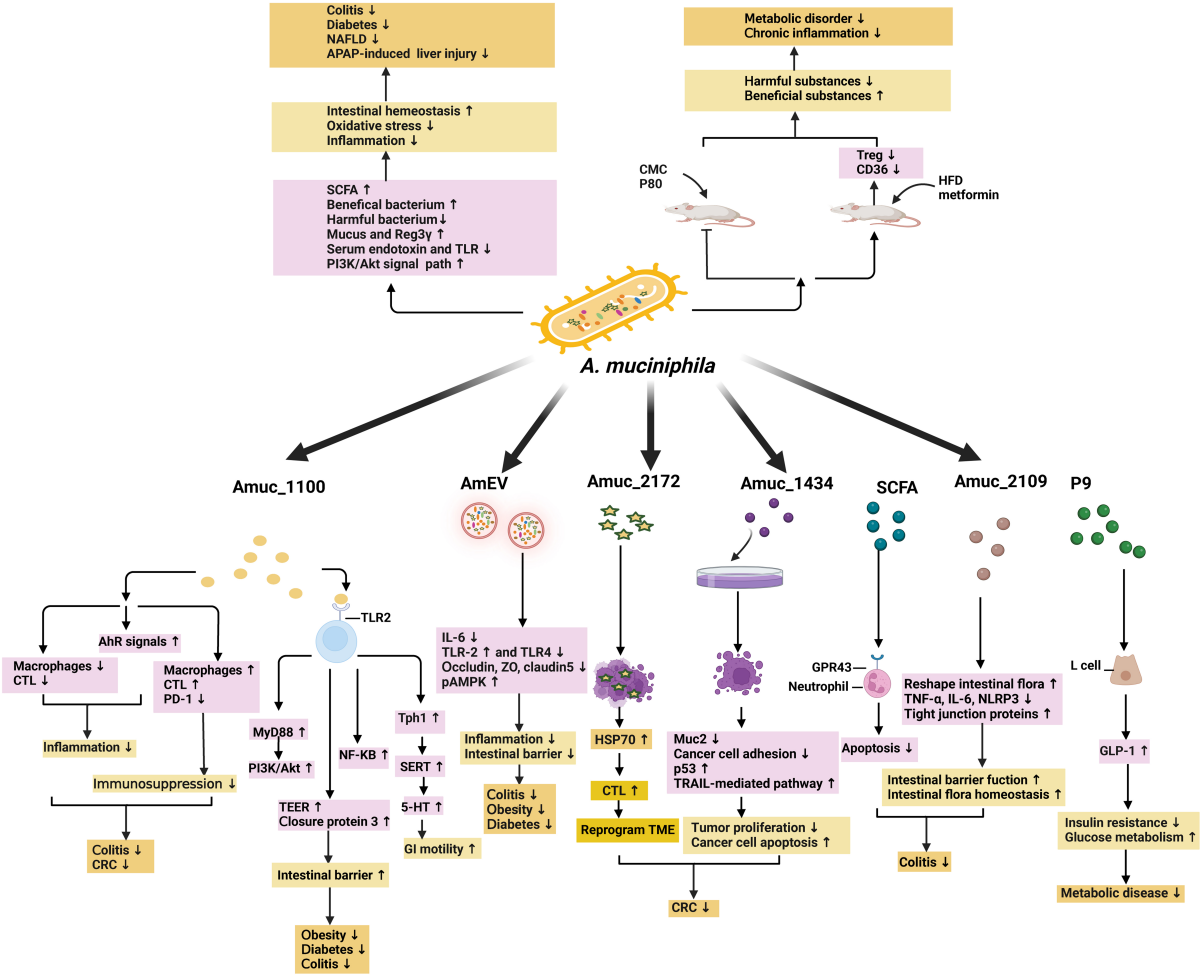
Diverse active components of *Akk* exert regulatory effects on intestinal-related diseases through distinct mechanisms. These findings offer insights into the potential use of these components as therapeutic agents in the clinical treatment of gastrointestinal disorders. Created with BioRender.com. *Akk*: *Akkermansia muciniphila*.

In recent years, a cancer vaccine preparation with extracellular vesicles mixed with lipid nanovesicles (Lipo@HEV) was constructed by using exosomes derived from tumor cells as antigen sources, and outer membrane vesicles from *Akk* (Akk-OMV) as natural adjuvants. It can enhance preventive and therapeutic vaccination by promoting the maturation of dendritic cells (DC) in lymph nodes and activating CTL reaction, and enhance the therapeutic effect of PD-1 blocking by loading PD-L1 trap plasmids^[41]^ [Figure 3].

### *Akk* and functional gastrointestinal disorders

*Akk* has also been reported to be associated with functional gastrointestinal disorders such as IBS. In 2012, a study discovered that the relative abundance of *Akk* in the feces of children with diarrhea-predominant irritable bowel syndrome was significantly reduced^[42]^.

Another study in 2017 found that the traditional Chinese medicine Wujiwan can effectively reduce abdominal pain and diarrhea in rats with post-inflammatory irritable bowel syndrome (PI-IBS), and its effect may be related to significantly improving the abundance of *Akk*^[43]^. Furthermore, fecal microbiota transplantation (FMT) was shown to effectively relieve gastrointestinal symptoms and relieve depression and anxiety in 30 patients with refractory IBS. The analysis of the fecal microbiota showed that the abundance of *Akk* was significantly increased one month after FMT^[44]^. Another study in 2018 also obtained similar results^[45]^ and found that the abundance of *Akk* is related to the extent of symptom relief of patients, and we speculate that the mechanism may lie in the decreased sensitivity of visceral pain caused by short chain fatty acid (SCFA), one of the metabolites of *Akk* and other probiotics^[46]^. Furthermore, in a clinical study, the researchers stimulated biopsied intestinal mucosa of PI-IBS and healthy people with *Akk* and found that the release of anti-inflammatory cytokine IL-13 in PI-IBS patients increased significantly after *Akk* stimulation, which was higher than that in the healthy control group, further explaining the anti-inflammatory effect of *Akk* in IBS^[47]^. In addition, a study assessing the changes of intestinal mucosa and microbiota associated with air pollution of UC and IBS patients in Ukraine found that the intestinal mucosa of patients in areas with low air pollutants particulate matter 2.5 (PM2.5) was less damaged than that in high PM2.5, and the level of *Akk* in feces of patients in low PM2.5 was even significantly higher than that in the healthy control group, further suggesting the protective effect of *Akk* in IBS^[48]^. In addition, several studies found that the relative abundance of *Akk* in the intestines of patients with constipation-predominant irritable bowel syndrome(C-IBS) increased^[49]^, which is different from previous studies finding that the abundance of *Akk* decreased in patients compared with the healthy population. However, Gobert *et al.*, using the model of human microbiota-related rats (HMAR) and experimental colitis induced by DSS, found that animals carrying C-IBS-related microbiota had milder colitis after DSS treatment^[18]^. Compared with the control group, in mice pretreated with *Akk* or HMAR, the expression of *IL-17*, *IFN-γ*, and *TNF-α* genes was inhibited, revealing that although the abundance of *Akk* in the intestines of C-IBS patients increased, its effect was still beneficial, that is, the anti-inflammatory effect of intestinal microbiota in C-IBS patients could have been mediated by *Akk* to some extent. In addition, another study on intestinal microbial changes in patients with enteritis and IBS shows that there is no significant difference in *Akk* abundance between IBS patients and the healthy population, which may be due to the limitation of sample size, because it only includes 3 IBS patients^[50]^. The above studies suggest the potential beneficial effects of *Akk* in IBS, but they are mostly association studies. There are limited studies using *Akk* to directly interfere with human or animal models to explore its role in IBS. Furthermore, whether *Akk* plays a harmful role in IBS in different models and the effects of *Akk* components in IBS are not fully understood, which warrants further exploration in the future.

### *Akk* and other intestinal-related diseases

#### Akk and metabolic diseases

*Akk* has a role in the prevention and treatment of diabetes, obesity, and other metabolic dysfunctions. In diabetes, the decreased expression of intestinal tight junction proteins leads to increased intestinal permeability, resulting in excessive absorption of lipopolysaccharides (LPS) and leading to chronic inflammation^[51]^. A clinical trial in 2016 showed that obese subjects with a higher abundance of intestinal *Akk* had healthier metabolic markers such as plasma triglycerides, fasting glucose, and body fat distribution^[52]^. In 2018, Hänninen *et al.* found the mice lacking *Akk* presented a high prevalence of autoimmune diabetes, also known as type I diabetes (T1D). Then, they transferred *Akk* experimentally to the mice with T1D and found that *Akk* transfer promoted mucus production and increased expression of antimicrobial peptide, as well as lowering serum endotoxin levels and islet Toll-like receptor (TLR) expression, which suggested that *Akk* plays a protective role in T1D^[53]^. The underlying mechanism may be that *Akk* will increase the production of anti-inflammatory cytokines in pancreatic lymph nodes and enhance the recruitment of Tregs in the pancreas, which will eventually delay the development of T1D^[54,55]^. *Akk* can also promote mucus production and increase the expression of antimicrobial peptides, reduce the level of serum endotoxin and the expression of islet TLRs, promote immune regulation, and delay the development of diabetes^[53]^.

The relative abundance of *Akk* is also significantly reduced during the development of type II^[56]^ diabetes. In addition, a study found that the feces of healthy individuals contained more AmEV compared to those with type II diabetes^[57]^. The purified membrane protein Amuc_1100 from *Akk* also improved metabolic disorders in obese and diabetic mice^[20]^. These studies provide strong evidence that *Akk* regulates the progress of diabetes. Furthermore, the abundance of *Akk* was also reduced in high-fat diet (HFD) mice and the metabolic disorders of HFD mice could be reversed through the supplementation of *Akk*^[57,58]^. In 2013, a study found that gavage of live *Akk* to HFD-induced obese mice reduced the level of plasma LPS by increasing intestinal barrier function, lowered the insulin resistance index, and normalized the adipose tissue marker CD11c, whereas heat-inactivated *Akk* could not exert such effects^[58]^. Yet another study^[20]^ in 2017 found that pasteurized *Akk* also significantly improved insulin sensitivity indices and that mice treated with pasteurized strains showed more significant reductions in body weight, plasma lipids, markers of insulin resistance, and blood inflammatory markers, compared with live bacteria^[20]^. There are many reasons that may lead to the opposite results; for example, the amounts of *Akk* used in the two experiments are different, and the medium used for bacterial culture is different. In addition, autoclaving *Akk* eliminated its beneficial effects. However, recent research^[59,60]^ showed that pasteurizing probiotics at 70 °C for 30 min, which is a less extreme treatment method to limit the degeneration of their cell components, can partially or completely retain its beneficial effects^[20]^. We speculate that the less intense heat inactivation, such as pasteurization, allows *Akk* to become more stable while retaining some of their beneficial properties, but the dead bacteria after complete thermal inactivation do not have this function.

Furthermore, *Akk* significantly reduced adipose inflammation by inducing more Foxp3^+^ regulatory T cells in obese mice^[61]^, *Akk* was significantly increased after treatment with metformin in obesity mouse model^[62]^, and higher levels of *Akk* in patients contributed to the efficacy of metformin^[63]^. It has been demonstrated that intestinal microbes such as *Akk* may modulate the efficacy of metformin through the production of SCFAs^[64]^. In 2019, Depommier *et al.* conducted a pilot study on the administration of *Akk* in overweight and obese human volunteers and found that oral administration of *Akk* for 3 months was completely safe and well tolerated by all volunteers. *Akk* significantly enhanced insulin sensitivity in obese volunteer patients, downregulated total cholesterol levels, and even improved liver tissue lesions, and the volunteers experienced substantial weight loss^[65]^. Furthermore, *Akk* has been shown to reduce gene expression involved in adipocyte differentiation and lipid oxidation in mouse mesenteric adipose tissue, suggesting its role in the regulation of lipid metabolism^[58]^ [Figure 2].

#### Akk and the “intestine-hepatic axis”

The liver is the detoxification organ of the body and is closely related to the intestine through the hepatic portal system. Thus, the occurrence of liver diseases such as hepatitis and hepatic steatosis is also associated with the intestine. The intestinal microbiota plays an important role in the interaction between the intestine and the liver. When the intestinal barrier is damaged, intestinal pathogenic bacteria and LPS translocate and enter the liver via the hepatic portal vein. Liver Kupffer cells phagocytose LPS and release cellular inflammatory factors, which damage liver function^[66]^. LPS further increases intestinal mucosal permeability, forming a vicious circle that may lead to the development of many liver and intestinal diseases. In this context, the application of probiotics such as *Akk* to improve the intestinal microenvironment may be effective in treating liver disease. A study using a streptozotocin high-fat diet (STZ-HFD) to induce mice model of liver disease showed that the relative abundance of *Akk* was reduced in the STZ-HFD group of mice compared to control mice^[67]^. In 2017, administration of *Akk* to experimental mice was demonstrated to reduce serum alanine transaminase and aspartate transaminase, attenuate liver damage induced by concanavalin A (Con A), and decrease both pro-inflammatory cytokines such as IL-2 and IFNγ in the serum of mice^[68]^. Consistent with the serological results, IFNγ expression was significantly reduced in the liver of *Akk*-treated mice^[68]^. In the same year, another study showed that rhubarb extract could prevent hepatitis caused by acute alcohol intake and increase the abundance of *Akk* in the intestine. Subsequent analysis revealed that the abundance of *Akk* was positively correlated with the expression of the antimicrobial peptide, regenerating islet-derived protein IIIγ (RegIIIγ), which restores the intestinal barrier function and prevents alcohol-induced liver injury. It is speculated that rhubarb extract may promote the expression of RegIIIγ by increasing the number of *Akk*^[69]^. In addition, it has been reported that berberine reduced acute-chronic alcoholic liver injury by altering the overall gut microbial community and promoting the abundance of *Akk*^[70]^. A study in 2018 found that *Akk* abundance in the feces of alcoholic hepatitis patients was lower than that of healthy individuals, and the abundance of *Akk* was significantly reduced by alcohol gavage in wild-type (WT) mice, while oral administration of *Akk* prophylactically reduced liver injury, fatty liver, and neutrophil infiltration^[71]^. Consistently, a clinical study showed that patients with either hepatocellular carcinoma or cirrhosis showed a decrease in *Akk* abundance, along with an increase in intestinal permeability and plasma LPS levels, suggesting that the hepatoprotective function of *Akk* may be achieved by reducing intestinal-derived inflammation^[72]^. Recently, it has been found that *Akk* can alleviate the hepatotoxicity induced by sodium valproate^[73]^, and oral application of *Akk* can alleviate the liver injury induced by alcohol, increase the serum ornithine level, and reduce the oxalic acid level increased by alcohol intake^[74]^. In addition, the imbalance of intestinal microbiota and the decrease of SCFA production in nonalcoholic steatohepatitis (NASH) animals may further act through the hepato-intestinal brain axis. It may lead to the exhaustion of dopamine in the frontal cortex, increasing protein oxidation and lipid peroxidation in the frontal cortex, which may eventually lead to mental illness. *Akk* has also been reported to reduce microglia proliferation and inflammation and improve cognitive impairment induced by NASH, including spatial working memory and new object recognition^[75]^. The deletion of NOD-like receptor family pyrin domain containing 6 (NLRP6) can enhance the progress of hepatitis, which may be related to the increase of *Muribaculum* and the decrease of *Akk* abundance, while *Akk* supplementation can improve intestinal barrier function, reduce the infiltration of myeloid-derived suppressor cells (MDSC), and inhibit steatohepatitis activity^[76]^. In addition, some studies have reported the correlation between Amuc_1100 and liver disease in mouse model. Amuc_1100 significantly reduces serum ALT and AST levels and the body weight of NAFLD mice, and ameliorates the blood lipid level. In the liver, *Akk* and Amuc_1100 significantly reduced the mRNA expression levels of NOD-like receptor thermal protein domain associated protein 3 (NLRP3) and TLR4/nuclear factor κB (NF-κB), as well as the protein and mRNA expression levels of inflammatory cytokines^[77]^. The above evidence suggests that supplementation with *Akk* may be a new effective approach for the prevention and treatment of liver injury [Figure 2].

## THE POTENTIAL MECHANISM OF *AKK* AND ITS EFFECTIVE COMPONENTS IN REGULATING INTESTINAL-RELATED DISEASES

In recent years, an increasing number of studies have focused on the potential mechanisms of the regulatory role of *Akk* and the postbiotics from *Akk*, such as pasteurized cells and the effective components or metabolites, aiming to provide probiotics in a more appropriate manner to promote the host health. The main potential mechanisms and their effective components are discussed herein [Table 1 and Figure 3].

**Table 1:** The mechanism of *Akk* and its effective components in intestinal-related diseases

**Bacterial components**	**Experimental model**	**Strain of *A. muciniphila***	**Target**	**Mechanism**	**Ref.**
Living *A. muciniphila*	DSS (mice)	139	Gut microbiota	Facilitate the normalization of the gut microbiota	[29]
	DSS (mice)	ATCC BAA835	Immunity	Promote the differentiation of Tregs and increase production of SCFAs	[29]
	DSS (mice)	ATTC BAA835	Gut barrier function and microbiota	Protect the gut barrier function, reduce the levels of inflammatory cytokines, and improve the microbial community	[30]
	*Salmonella pullorum* (chicken)	ATTC BAA835	Gut barrier function and microbiota	Downregulate the abundance of *S. pullorum* and accelerate the proliferation of intestinal epithelium through the Wnt/β-catenin signaling pathway	[78]
	DSS (mice) treated with *L. pentosus*	Unclear	Gut microbiota	Collaborate to produce metabolites beneficial to regulating intestinal immunity	[79]
	NOD (mice)	CIP 107961T	Gut microbiota and immunity	Induce gut microbiota remodeling, promote mucus and RegIIIγ production, and decrease the levels of serum endotoxin and islet TLR expression	[53]
	HFD (mice) treated with quercetin	CIP-107961T	Gut microbiota and bile acid	Enhance the abundance of *Cyanobacterium* and *Oscillospira* and BA synthesis	[80]
	APAP-mediated hepatotoxicity (mice)	ATTC BAA835	Gut microbiota and immunity	Attenuate APAP-induced oxidative stress and the inflammatory response and remodel the gut microbiota	[81]
	Chronic intestinal inflammation induced by CMC and P80 (mice)	ATTC BAA835	Gut microbiota	Prevent the destruction of CMC and P80 on intestinal microbiota and change of the colon gene expression	[82]
	HFD (mice) treated with metformin	CIP 107961T	Immunity	Enhance glucose tolerance and reduce inflammation of adipose tissue by inducing Tregs in visceral adipose tissue	[61]
	HFD (mice) treated with D3	ATCC BAA-835	Gut microbiota	Inhibit lipid absorption by downregulating the expression of CD36 and modulating the gut microbiota	[83]
Pasteurized *A. muciniphila*	HFD (mice)	ATTC BAA-835	Gut barrier function	Increase lysozyme Lyz1 expression to enhance intestinal barrier function	[20]
	DSS (mice)	ATCC BAA-835	Immunity	Reduce the infiltration of pro-inflammatory macrophages and CTL in the spleen and intestinal lymph nodes of mice with colitis	[21]
	AOM/DSS (mice)	ATCC BAA-835	Immunity	Increase the number of CTL in MLN and inhibit the expression of PD-1 on pro-inflammatory macrophages of MLN and spleen, and reduce the proportion of PD-1^+^CTL	[21]
	HFD/CCl4 (mice)	ATCC BAA-835	Gut barrier function and immunity	Decrease the expression of *TLR-2*, *TLR-4* and *TNF-α* genes, and enhance the expression of tight junction protein	[6]
AmEVs	DSS (mice)	ATCC BAA-835	Immunity	Reduce the production of pro-inflammatory cytokine IL-6 in colon epithelial cells induced by *Escherichia coli* EV	[14]
	HFD (mice)	ATCC BAA-835	Immunity	Reduce the expression of TLR-4 and TLR-2 in colon	[84]
	HFD (mice)	ATCC BAA-835	Gut barrier function	Enhance intercellular tight junctions by upregulating the expression of occludin, ZO, and claudin-5	[84]
	HFD (mice)	ATCC BAA-835	Gut barrier function	Improve intestinal barrier integrity by inducing AMPK phosphorylation	[57]
Amuc_1434	Colon cancer cell line LS174T	ATCC BAA-835	Cell proliferation	Upregulate the expression of p53 and result in the arrest of CRC cells in the G0/G1 phase of the cell cycle	[40]
	Colon cancer cell line LS174T	ATCC BAA-835	Cell adhesion	Bind with normal colon cells and degrade Muc2 secreted by colon cancer cells, as well as promoting the mutual adhesion of colon cancer cells with high Muc2 expression	[85]
Amuc_1100	HFD (mice)	ATCC BAA-835	Gut barrier function	Interact with TLR2 on the cell surface to improve the intestinal barrier function	[20]
	PBMC	ATTC BAA-835	Immunity	Bind TLR2 or TLR4 on PBMCs and activate the NF-κB signaling pathway to produce high levels of IL-10	[86]
	DSS (mice)	ATCC BAA-835	Immunity	Reduce the infiltration of pro-inflammatory macrophages and CTL in the spleen and intestinal lymph nodes of mice with colitis	[21]
	AOM/DSS (mice)	ATCC BAA-835	Immunity	Increase the number of CTL in MLN and inhibit the expression of PD-1 on pro-inflammatory macrophages of MLN and splenic, and reduce the proportion of PD-1^+^CTL	[21]
	DSS (mice)	Unclear	Trp metabolism	Increase the expression of AhR-targeted genes such as *IL-10* and *CYP1A1*	[87]
	Caco-2 cells	ATCC BAA-835	5-HT	Promote the expression of 5-HT synthesis rate-limiting enzyme Tph1 in RIN-14B cells and reduce the expression of SERT in Caco-2 cells through the direct interaction with TLR2	[88]
Amuc_2172	AOM/DSS (mice)	ATCC BAA-835	Immunity	Acetylate the Lys14 site on histone H3 of the HSP70 gene to promote HSP70 secretion and activate CTLs	[4]
	CT26 bearing (mice)				
	*Apc*^min^/^+^ (mice)				
Amuc_2109	DSS (mice)	Unclear	Gut microbiota and immunity	Reshape the intestinal microbiota and inhibit the expression of the pro-inflammatory factors and NLRP3	[89]
P9	HFD (mice)	ATCC-BAA-835	Immunity	Stimulate the expression of IL-6 and promote the secretion of GLP-1 through ICAM2	[90]

*Akk/A. Muciniphila*: *Akkermansia muciniphila*; DSS: dextran sulfate sodium salt; Tregs: regulatory T cells; SCFA: short chain fatty acid; NOD: nucleotide-binding oligomerization domain; RegIIIγ: regenerating islet-derived protein IIIγ; TLR: Toll-like receptor; HFD: high-fat diet; BA: bile acids; APAP: acetaminophen; CMC: carboxymethyl cellulose; P80: polysorbate 80; Lyz1: lysozyme 1; CTL: cytotoxic T lymphocyte; AOM: azoxymethane; MLN: mesenteric lymph nodes; PD-1: programmed death 1; TNF-α: tumor necrosis factor-α; AmEVs: extracellular vesicles of *Akk*; IL-6: interleukin-6; EV: extracellular vesicles; ZO: zonula occludens; AMPK: adenylate-activated protein kinase; CRC: colorectal cancer; PBMC: peripheral blood mononuclear cell; NF-κB: nuclear factor kappa-light-chain-enhancer of activated B cells; Trp: tryptophan; AhR: aryl hydrocarbon receptor; 5-HT: 5-hydroxy tryptamine; Tph1: tryptophan hydroxylase 1; HSP70: heat shock protein 70; NLRP3: NOD-like receptor thermal protein domain associated protein 3; P9: protein 9; GLP-1: glucagon-like peptide-1; ICAM2: intercellular adhesion molecule-2.

### The effect of living *Akk*

The development of intestinal diseases is often accompanied by dysbiosis and disturbance of the intestinal microbiota^[91]^. Studies have shown that intestinal probiotics tend to alleviate intestinal diseases by regulating intestinal homeostasis, inhibiting the growth of harmful bacteria, and promoting the reproduction of beneficial bacteria. *Akk* gavage has been found to increase the production of the metabolite SCFAs such as acetate and propionate, restore the damaged intestinal microbiota, and alleviate colonic inflammation in mice^[29,30]^. In addition, *Akk* inhibits the growth of *Salmonella pullorum*, thereby reducing colonic mucosal damage^[92]^. Moreover, *Akk* was able to interact with other intestinal bacteria to regulate intestinal immune function and alleviate colonic inflammation. In DSS-induced colitis mice, gavage of *Lactobacillus pentosus* significantly increased the abundance of *Akk* and made *Akk* the dominant bacterium in the colon, which reduced colonic inflammation^[79]^. In another study, the administration of *Akk* to DSS-induced colitis mice increased the abundance of *Clostridia*, *Firmicutes*, *Ruminococcaceae*, and *Akkermansia*, and inhibited *Bacteroidetes*, further demonstrating that *Akk* reshapes the gut microbial community^[30]^. Additional studies have observed that *Akk* induces gut microbiota remodeling and controls islet autoimmunity in non-obese diabetic mice, potentially reducing the incidence of diabetes^[53]^. *Akk* promoted mucus production and the expression of the antimicrobial peptide RegIIIγ, decreased the abundance of *Ruminococcus* and the levels of serum endotoxin, as well as islet TLR expression, thus inhibiting the development of diabetes. It was also found that the combination of *Akk* and quercetin could drive protective effects against obesity and NAFLD through modulation of the gut microbiota^[80]^. In addition, recent studies have discovered the potential mechanism of *Akk* regulating intestinal microbial composition. Firstly, *Akk* can support the growth of butyrate producers by degrading mucin and producing acetate and propionate^[93,94]^. Secondly, *Akk* can use the vitamin B12 produced by other bacteria to produce propionate^[95]^. Furthermore, another study has shown that *Akk* can produce sulfatase, thus producing cysteine by hydrogen sulfide, which may alleviate the toxicity caused by sulfate-reducing bacteria in the host^[96]^. Taken together, the regulation of intestinal microbiota homeostasis is also an important part of the role of *Akk per se*.

In addition to directly inhibiting or promoting other bacteria, *Akk* also plays a beneficial role in intestinal-related diseases by inhibiting the toxicity of harmful substances or promoting the effect of beneficial substances^[82,83,97]^. Increasing evidence showed that some unabsorbed food additives, including the emulsifier carboxymethyl cellulose (CMC) and polysorbate 80 (P80), can cause the destruction of the microbial community, such as the depletion of *Akk* and the following chronic intestinal inflammation. Supplementing exogenous *Akk* to mice can prevent the harmful effects of emulsifiers, such as hyper appetite, weight gain, and blood glucose abnormalities. The administration of *Akk* also alleviated the slight intestinal inflammation induced by CMC and P80^[82]^. Additionally, *Akk* supplementation can protect the intestinal microbiota from the damaging effects of CMC and P80^[82]^. It is noteworthy that the study discovered that CMC and P80 changed inflammation-related gene expression in the colon, which was prevented by *Akk*. Moreover, it has been reported recently^[97]^ that the colonization of *Akk*. in the intestine can increase the expression of intestinal cAMP reactive element-binding protein H (CREBH), thus promoting the production of intestinal barrier tight junction protein. In addition, *Akk*. promotes the binding of CREBH to microRNA-143/145 (miR-143/145), thus promoting intestinal epithelial cell (IEC) regeneration and wound repair through insulin-like growth factor (IGF) and IGF-BP 5 signal transduction^[97]^. The Amuc_1100 secreted by *Akk* can also cover this function of *Akk cells* in IECs^[97]^. Another study designed a peptide of 9 amino acids named D3, which is a novel drug candidate for inhibiting diet-induced obesity as a non-toxic and bioactive peptide. It was found that after administration of D3, the abundance of intestinal *Akk* in mice increased by 100 times, which inhibited lipid absorption by downregulating the expression of CD36, thus reducing diet-induced obesity^[83]^.

### The effect of AmEVs

The extracellular vesicles derived from probiotics are also beneficial in intestinal-related diseases^[84,98]^, and AmEVs showed a similar effect as *Akk* in colitis mouse model induced by DSS^[57]^. AmEVs significantly decreased in the feces of obese patients and DSS-induced colitis mice. The application of AmEVs reduced the weight gain and fat content induced by HFD, and lowered the production of IL-6 in colon epithelial cells. Moreover, oral gavage of AmEVs reduced DSS-induced colitis by alleviating weight loss, colon length reduction, and inflammatory cell infiltration. The underlying mechanism is that the administration of AmEVs significantly reduced the expression of TLR4 and activated TLR2 in the colon of obese mice^[84]^. On the other hand, AmEVs have been found to enhance intercellular tight junctions by upregulating the expression of occludin, ZO, and claudin-5. In liver diseases, AmEVs prevented chemical-induced liver injury in mice via normalizing fecal bacterial composition, reducing intestinal permeability, and inhibiting inflammatory reactions^[6]^. Chelakkot *et al.* found that the administration of AmEVs reduced weight gain and improved glucose tolerance in HFD-induced diabetic mice^[57]^ and illustrated the underscoring mechanism that AmEVs improved intestinal barrier integrity in HFD-induced diabetic mice by inducing adenylate-activated protein kinase (AMPK) phosphorylation, which increased the expression of tight junction proteins in IECs. However, it is noteworthy that the administration of AmEVs reduced the daily food intake of HFD mice, which may be another important factor in improving metabolism besides its own direct effect. In addition, the dose of AmEVs in the experiment is not clear. Despite the aforementioned beneficial effects of AmEVs in intestinal related disease, the components in AmEVs remain unclear and whether these substances mediate the function of AmEVs in intestinal related disease requires further study.

### The effect of Amuc_1434

Recent studies have focused on the mechanism of specific components from *Akk*, such as various proteins, in intestinal-related diseases. It was found that *Akk* can directly promote apoptosis of tumor cells, and this effect was associated with Amuc_1434, a protein from AmEV. Amuc_1434 binds with normal colon cells and degrades Muc2 secreted by colon cancer cells. Amuc_1434 also promotes the mutual adhesion of colon cancer cells with high Muc2 expression^[99]^. Subsequently, Amuc_1434 was shown to upregulate the expression of p53, an oncogene that controls cell cycle initiation, which resulted in the arrest of CRC cells in the G0/G1 phase of the cell cycle and inhibited the proliferation of colon cancer cells. Furthermore, Amuc_1434 also induced apoptosis in CRC tumor cells by activating the mitochondrial apoptosis pathway associated with TNF-related apoptosis-inducing ligand^[40]^.

### The effect of Amuc_1100

Another protein from *Akk* is Amuc_1100, which was widely studied and purified from the outer membrane of *Akk*. Amuc_1100 was isolated and identified for the first time in 2017, and it was demonstrated to improve the metabolism of obese and diabetic mice^[20]^. The study investigated the potential mechanism of the protective role of Amuc_1100, and confirmed that Amuc_1100^[20]^ was associated with the formation of *Akk* pili and was capable of interacting directly with TLR2. Activated TLR2 increased the expression of tight junction protein between IECs, and enhanced the anti-apoptosis ability of IECs mediated by phosphatidylinositol 3 kinase/protein kinase B (PI3K/Akt) pathway through myeloid differentiation factor 88 (MyD88). Furthermore, it was also found that Amuc_1100 and LPS from *Akk* all bound TLR2 or TLR4 on peripheral blood mononuclear cells (PBMCs) and activated the NF-κB signaling pathway to produce high levels of IL-10, thereby regulating the host immune response^[86]^. In addition, another study in 2020 further investigated the mechanism of Amuc_1100 in intestinal-related diseases, which found that pasteurized *Akk* or Amuc_1100 can reduce the infiltration of pro-inflammatory macrophages and CTL in the spleen and MLN of mice with colitis, reduce histological damage in the proximal colon, and alleviate colitis^[21]^. In addition, it was found that pasteurized *Akk* or Amuc_1100 attenuated DNA damage, reduced apoptosis, and abnormal proliferation in colonic epithelial cells of colitis-associated CRC mice induced by AOM/DSS. Moreover, *Akk* or Amuc_1100 significantly increased the number of CTL in MLN and inhibited the expression of PD-1 on pro-inflammatory macrophages and CTL of MLN and spleen^[21]^, which effectively increased the immune effect of the host against CRC. Furthermore, a study in 2021 found that Amuc_1100 can increase the expression of aryl hydrocarbon receptor (AhR)-targeted genes such as *IL-10* and *CYP1A1* to alleviate colonic inflammation by regulating tryptophan metabolism and activating AhR signaling^[100]^, and this is a novel mechanism by which Amuc_1100 relieved intestinal inflammation. Another study in 2021 found that Amuc_1100 can promote the expression of 5-hydroxytryptamine (5-HT) synthesis rate-limiting enzyme Tph1 and reduce the expression of 5-HT reuptake transporter (SERT) in Caco-2 cells through the direct interaction with TLR2, thus improving the biosynthesis and level of 5-HT. As 5-HT is a neurotransmitter and an important signal molecule for regulating gastrointestinal function, Amuc_1100 can improve the gastrointestinal peristalsis function of mice by regulating the biosynthesis of 5-HT^[101]^. Although there is no research about the role of Amuc_1100 in IBS, we still infer from this research that Amuc_1100 may have the potential to improve IBS, and this effect may be related to 5-HT.

### The effect of Amuc_2172

Amuc_2172 is a newly discovered protein derived from *Akk*, which is a probiotic enzyme secreted by bacteria that can improve the function of host cells and has been shown to modulate the tumor immune microenvironment and thus inhibit a variety of CRC models and other tumor models such as melanoma. The researchers used proteases to identify Amuc_2172 as a protein from AmEVs. The mechanism of its tumorigenesis inhibition is that Amuc_2172, as a prokaryotic-derived acetyltransferase, can enter colorectal cells via macropinocytosis and acetylate the Lys14 site on histone H3 of the *HSP70* gene, thus promoting HSP70 secretion, activating CTLs, and promoting IFN-γ expression to play a role of tumor-killing^[4]^. The application of macrophage membrane-coated Amuc_2172 nanoparticles can promote the targeted delivery of Amuc_2172 to tumor cells, thus enhancing its antitumor effect. Importantly, it was found that Amuc_1100 protein was not detected in AmEVs and that Amuc_2172 had more potential to activate CTL compared to Amuc_1100. In addition, the results show that the mechanism of activating CTL by Amuc_1100 and Amuc_2172 may be different, because CTL can be activated by Amuc_1100 directly, while the activation of CTL by Amuc_2172 requires the involvement of cancer cells. Specifically, Amuc_2172 promotes the secretion of HSP70 from cancer cells, which in turn indirectly promotes the activation of CTL. However, whether Amuc_2172 can inhibit the development of intestinal-associated metabolic diseases as Amuc_1100 has not been reported and the mechanism of Amuc_2172 in intestinal-related diseases still needs further exploration. Altogether, as a probiotic enzyme, Amuc_2172 could regulate the function of eukaryotic cells and serve as a potential drug by catalyzing eukaryotic protein targets.

### The effect of other components

In addition to the above components, other active ingredients of *Akk* are also important in intestinal-related diseases. Pasteurized *Akk* can alleviate the progress of intestinal-related diseases by enhancing intestinal barrier function and regulating intestinal immunity, as mentioned above^[21]^. The metabolite of *Akk*, SCFA, can act on G-protein-coupled receptor 43 (GPR43). Compared with WT enteritis mice, Gpr43^-^/^-^ enteritis mice showed higher expression of IL-6 and IL-22 and more tissue damage. After SCFA supplementation, it binds to GPR43 on neutrophils and subsequently induces their apoptosis, which alleviates the progression of enteritis in WT mice^[102]^, whereas no remission was seen in Gpr43^-^/^-^ mice. SCFA can also affect glucose metabolism to improve type II diabetes, and it regulates intestinal microbial composition and metabolism to inhibit liver injury^[81,103]^. Moreover, a recent study found that another probiotic enzyme Amuc_2109, a metabolic enzyme (β- N-acetyl hexosaminidase) secreted by *Akk*, had a significant protective effect against DSS-induced colitis mainly by improvement of the intestinal epithelial barrier function and regulation of intestinal microbiota homeostasis^[89]^, as Amuc_2109 reshaped intestinal microbiota and inhibited the overexpression of TNF-α, IL-6, and NLRP3 in DSS-induced colitis, as well promoting the expression of tight junction protein. However, Amuc_2109 was not detected in *Akk* grown on mucin^[104]^; its role in intestinal-related diseases needs to be further explored. Furthermore, a recent study identified a new protein secreted by *Akk*, named protein 9 (P9), which stimulates human intestinal endocrine L cells to secrete glucagon-like peptide-1 (GLP-1) *in vitro* and causes a modest increase in circulating GLP1 levels of obese mice, thereby reducing insulin resistance and improving glucose metabolism. The process may be associated with the activation of intercellular adhesion molecule 2 (ICAM2), as the ICAM2 antibody partially abrogated the effect of P9 on GLP1 *in vitro*^[90]^. Moreover, the expression of ICAM2 may require the involvement of IL-6. The study illustrates the new mechanism by which *Akk*-derived proteins act on intestinal endocrine cells to affect the occurrence and development of metabolic diseases. However, the signal pathways through which P9 protein interacts with ICAM-2, and the downstream signal transduction through which ICAM-2 induces GLP-1 secretion are unclear, and the specific molecular mechanism still needs to be explored by more experiments. In addition, ornithine lipid, a lipid component produced by *Akk* in the intestines of mice and humans, was also reported to have anti-inflammatory effect, which can prevent LPS-induced inflammatory reaction, inhibit the production of pro-inflammatory cytokines, and increase the level of anti-inflammatory cytokine IL-10^[105]^.

## PATHOGENESIS OF *AKK* IN INTESTINAL-RELATED DISEASES

The above research shows the beneficial effect of *Akk* in intestinal-related diseases. On the other hand, some studies have also found that the application of *Akk* aggravated intestinal inflammation in mice. A study in mice with secondary metastatic colitis (AdTr-colitis) also found that the abundance of *Akk* was positively correlated with the degree of colonic inflammation and histopathological score^[106]^. In 2013, the results of a study showed that commensal *Akk* exacerbated intestinal inflammation in *Salmonella typhimurium*-infected germ-free mice^[107]^, and then in 2017, Seregin *et al.* reported that repeated gavage of *Akk* could induce increased severity of colitis in IL10^-^/^-^ mice^[108]^. There are many possible reasons for the controversial role of *Akk* in intestinal inflammation. These include the use of various mouse models in different experiments - some are DSS-induced mouse models and some are knockout mouse models - and the different amounts of *Akk* administrated. In addition, there are differences in the specificity of the strains used: different strains of the same species may have opposite effects in the same disease. Most current studies on *Akk* are limited to the model strain isolated in 2004, ATCC BAA-835T. However, another newly isolated strain of *Akk* in 2017 has been shown to exacerbate colitis in IL-10^-^/^-^ mice. In addition, a study in 2019 reported differences in anti-inflammatory function between *Akk* BAA-835T and mouse-derived *Akk* strain 139. Therefore, the strain specificity of *Akk* may also be the main reason for the different results of different experiments. The pro-inflammatory effect of lipid components of *Akk* has recently been discovered. As membrane phospholipids from *Akk* can induce BMDCs to produce pro-inflammatory cytokines such as TNF-α in a TLR2-dependent manner^[109]^. Furthermore, similar to intestinal inflammation, the role of *Akk* in CRC is controversial. A recent study in 2022 found that receiving *Akk* administration exacerbated the development of colitis-associated CRC in AOM/DSS-induced model mice, as evidenced by shorter colon length, more severe weight loss, and more intestinal tumors^[110]^. There are multiple possible reasons to explain the differences in the results of different experiments. First, the viability of *Akk*, the frequency of administration, and the number of bacteria, all affect *Akk* colonization and CRC development. Furthermore, the role of *Akk* may be different in different CRC models. The classical AOM/DSS-induced CRC model was chosen in this study in 2022, which may be different from the spontaneous CRC models such as *APC^min^/^+^* mice in other studies. More studies are required in the future to explore and confirm the role of *Akk* in intestinal tumors and intestinal inflammation. In addition, future studies on *Akk* should pay detailed attention to the strains and evaluation of the probiotic effect of each type of *Akk* strain. So far, there is still a lack of relevant research on whether *Akk* has adverse effects on intestinal functional diseases and metabolic diseases. In this context, the probiotic role of *Akk* in intestinal-related diseases and its specific mechanisms need to be explored further in the future.

## CONCLUSION AND PERSPECTIVE

In summary, as a paradigm in the new generation of probiotics, the colonization of *Akk* in the intestine is closely related to maintaining intestinal homeostasis. It plays a critical role in the progression and treatment design of intestinal inflammation, cancer, and other diseases caused by intestinal disorders, such as liver diseases and diabetes. While the majority of research indicates the beneficial effects of *Akk* treatment, some studies suggest it may exacerbate disease progression. This discrepancy could be attributed to variations across studies, such as differing disease models, the distinct intestinal environments in which *Akk* operates, the activity levels of various *Akk* strains, or the specific components of *Akk* used. It is worth mentioning that the extracellular vesicles and proteins of *Akk* are increasingly well studied, and studies have shown that these components may be more protective when used alone than active organisms, which is more suitable for clinical applications. In addition, pasteurized intact *Akk* cells are considered safe for human consumption and thus are suitable for clinical application. They have been shown to be as effective or even more effective than living cells in the treatment of metabolic disorders. Therefore, it is of interest to continue exploring the pasteurized cells, extracellular vesicles, and bacterial outer membrane fractions of *Akk* to extract and utilize the fractions that play an important role. Furthermore, studies on the brain-gut axis and lung-gut axis have been emerging in recent years, and in addition to metabolic and liver diseases, the association between intestinal homeostasis and other systemic diseases is gaining more attention, and future studies may explore the role of *Akk* in other diseases in the future. In conclusion, as a potential probiotic, *Akk* is stably colonized in the intestine, and there is substantial evidence of its positive effects on intestinal-related diseases. Further evaluation of *Akk*’s safety, or the development of its active ingredient for targeted drug delivery, could pave the way for its clinical application as a therapeutic agent. Additionally, probiotic enzymes could be defined as prokaryotic isoenzymes from intestinal bacteria, which catalyze eukaryotic targets from host cells and function as potential drugs. As probiotic enzymes from *Akk*, both Amuc_2172 and Amuc_2109 could be potential drugs for colitis and inspire novel strategies in the treatment of intestinal-related diseases.

## DECLARATIONS

### Availability of data and materials

All data relevant to the review are included in the article.

### Copyright

© The Author(s) 2024.
